# ‘Is it better not to know certain things?’: views of women who have undergone non-invasive prenatal testing on its possible future applications

**DOI:** 10.1136/medethics-2018-105167

**Published:** 2019-01-24

**Authors:** Hilary Bowman-Smart, Julian Savulescu, Cara Mand, Christopher Gyngell, Mark D Pertile, Sharon Lewis, Martin B Delatycki

**Affiliations:** 1 Murdoch Children’s Research Institute, Parkville, Victoria, Australia; 2 Faculty of Medicine, Nursing and Health Sciences, Monash University, Clayton, Victoria, Australia; 3 Uehiro Centre for Practical Ethics, University of Oxford, Oxford, UK; 4 Department of Paediatrics, The University of Melbourne, Parkville, Victoria, Australia; 5 Victorian Clinical Genetics Services, Parkville, Victoria, Australia

**Keywords:** genetic counselling/prenatal diagnosis, genetic screening/testing, genetic selection, obstetrics and gynaecology, predictive genetic testing

## Abstract

Non-invasive prenatal testing (NIPT) is at the forefront of prenatal screening. Current uses for NIPT include fetal sex determination and screening for chromosomal disorders such as trisomy 21 (Down syndrome). However, NIPT may be expanded to many different future applications. There are a potential host of ethical concerns around the expanding use of NIPT, as examined by the recent Nuffield Council report on the topic. It is important to examine what NIPT might be used for before these possibilities become consumer reality. There is limited research exploring views of women on possible future uses of NIPT, particularly those of women who have undergone NIPT. In this study, we examined the views of women who undertook NIPT previously on the acceptability of and interest levels in using NIPT for a number of current and possible future applications. These included several medical conditions encompassing psychiatric, neurodevelopmental and adult-onset conditions as well as non-medical traits such as intelligence. One thousand women were invited to participate and 235 eligible surveys were received. Women generally reported an interest in using NIPT for medical conditions that severely impacted quality of life and with an onset earlier in life and stressed the importance of the accuracy of the test. Concerns were raised about the use of NIPT for non-medical traits. Respondents indicated that termination of pregnancy was not their only reason for testing, particularly in the case of sex. These results can further inform the ethical debate around the increasing integration of NIPT into healthcare systems.

## Introduction

Non-invasive prenatal testing (NIPT) is rapidly supplanting previously established methods of prenatal screening for chromosomal anomalies such as combined first trimester screening (CFTS).^1^ NIPT isolates and analyses cell-free fetal DNA circulating in the maternal bloodstream. Currently, NIPT screens for chromosomal conditions such as trisomy 21 (Down syndrome), sex chromosome aneuploidies (SCAs) and in some cases, copy number variants.^2^ The use of NIPT may be expanded further in future, with whole-genome sequencing of the fetus a possibility.^3^

NIPT is more accurate than CFTS, with very high sensitivity (99.3%) and specificity (99.9%) for trisomy 21.^4 5^ NIPT is safer than invasive diagnostic techniques such as chorionic villus sampling and amniocentesis, which carry a miscarriage risk of 0.1%–0.2%.^6^ However, NIPT is not diagnostic and therefore a high-risk result should be followed by invasive testing for confirmation.

NIPT can also be performed earlier in the pregnancy than CFTS (which includes results from the 12 week ultrasound) and requires only a maternal blood sample. It is currently available in Australia solely through the private sector, although it is being implemented in some other public healthcare systems such as the NHS in the UK.^7^

A 2017 Nuffield Council on Bioethics report made recommendations on the implementation of NIPT in the UK and discussed relevant ethical issues. Limits recommended in this report included restrictions on some practices which are already routine in the Australian setting, such as use of NIPT for fetal sex determination.^7^ A 2015 statement from the European and American Societies of Human Genetics advocated a cautious approach to future uses, recommending possible expansion to target ‘serious’ congenital and childhood disorders.^8^

Views of various stakeholders regarding implementation of NIPT have previously been investigated, with several studies focused on women who have undergone NIPT.^9–14^ However, there has been limited insight into the views of women on its possible future uses. The limited literature available has assessed the attitudes of pregnant women and focused on the future use of NIPT in terms of severity and timing of onset of a condition.^15–18^ This evidence suggests that there is a moderate amount of support in screening for adult-onset conditions, such as hereditary cancers. Research on views towards a broader suite of traits and conditions is lacking. In this study, we aimed to evaluate the attitudes of women who have used NIPT in Victoria, Australia towards the use of NIPT for a variety of conditions and traits, including current and possible future uses.

## Methods and materials

### Survey

A survey was developed to assess attitudes towards NIPT. The survey was developed in conjunction with professionals with relevant expertise in clinical genetics, bioethics, moral psychology, laboratory science and public health. For 14 traits or conditions, 5-point Likert scales were developed, and respondents were asked whether they (1) think NIPT should be available for that trait/condition, (2) would use NIPT to screen for that trait/condition and (3) would terminate an affected pregnancy. Some of the 14 conditions and traits can be tested for by NIPT currently, some are likely to be possible in the future, while some are more hypothetical. The 14 conditions and traits are listed in table 1. The complexity of the genotype-phenotype associations for several of the conditions and traits was noted. The survey can be found in the online supplementary material.

10.1136/medethics-2018-105167.supp1 Supplementary file 1

**Table 1:** Traits and conditions included in survey

Trait	Currently screened for with NIPT	Clinical relevance	Timing
Sex	Yes	Not clinically relevant	N/A
Trisomy 21	Yes	Intellectual, physical	Congenital
Trisomies 13 and 18	Yes	Intellectual, physical	Congenital
Mental illness	No	Psychiatric	Mixed
Non-medical traits such as increased or decreased intelligence	No	Not clinically relevant	N/A
Low-functioning autism	No	Psychiatric, intellectual, developmental	Mixed
High-functioning autism	No	Psychiatric, intellectual, developmental	Mixed
Impulse control disorders such as attention deficit hyperactivity disorder (ADHD)	No	Psychiatric, developmental	Mixed
Antisocial traits	No	Psychiatric	Mixed
Sex chromosomal aneuploidies with 20% decrease in intelligence quotient (IQ) and normal fertility	Yes	Intellectual	Congenital
Sex chromosomal aneuploidies with 10% decrease in IQ and infertility	Yes	Intellectual, fertility	Congenital
Deafness	No	Physical	Congenital/early
Preventable adult-onset conditions such as heart disease or cancer	No	Physical	Adult
Non-preventable adult-onset conditions such as early-onset Alzheimer disease	No	Physical, degenerative	Adult

These conditions/traits were selected based on several factors, including clinical relevance and timing (table 1). The survey also included sections to determine the respondent’s demographic characteristics as well as their religious and ethical beliefs using the Centrality of Religiosity scale^19^ and the Oxford Utilitarianism Scale,^20^ respectively. Questions with free-text responses were also included. Another section was included to assess respondents’ experiences with NIPT (data not included herein).

### Recruitment

Invitations to complete the survey were mailed to 1000 women randomly selected from the women who had undergone the *percept* NIPT test through the Victorian Clinical Genetics Services in 2016, out of 14 860 referrals. *Percept* screens for all autosomal trisomies and SCAs. It also automatically determines the sex of the fetus, although the parents can choose whether or not to have this disclosed to them by their healthcare practitioner. It is not covered by government rebate and therefore the cost is fully covered by the user. In this user-pays context, high-risk indications are not required for a referral.

Surveys were anonymous and could be completed online or using a hard copy provided in a follow-up reminder letter sent out approximately 2 weeks following first contact.

### Analysis

Data were analysed using STATA IC 15.1 (Stata, College Station, Texas, USA) and NVivo 12 (QSR International). Preliminary descriptive analyses were used to generate frequency data to produce a description of respondents. Differences between responses were assessed using two-sided paired sign tests. A p value of less than 0.05 was deemed statistically significant. For data presentation and sign tests, the 5-point Likert scales (Definitely not, Probably not/Unlikely, Unsure/Neutral, Probably/Likely, Definitely) were collapsed to 3-point scales resulting in groupings of positive, unsure or negative responses. Associations were analysed by ordinal logistic regression, presented with ORs and Bonferroni corrections applied. Analysis of qualitative free-text responses was performed independently by two researchers (CM and HBS) in NVivo using thematic analysis.^21^ Quotes are presented with an assigned number for each respondent.

## Results

The total number of surveys received was 237. Two were excluded as the respondent did not specify their age and were therefore ineligible to participate. The eight incomplete survey responses were included. Respondent demographics are seen in table 2.

**Table 2:** Demographic features of the cohort

	Respondents (n)	Percentage
Age (n = 235)		
18–25	0	0
26–30	23	9.8
31–35	93	39.6
36–40	79	33.6
41+	40	17
Result (n = 232) *		
No increased risk	227	97.8
Increased risk of trisomies 13, 18 or 21†	3	1.3
Increased risk for a sex chromosome aneuploidy‡	1	0.4
Other‡	1	0.4
Highest level of education (n = 225)		
Primary school	0	0
Secondary school	19	8.4
Technical or trade certificate	27	12
Bachelor’s degree	106	47.1
Postgraduate qualification (eg, Masters, PhD)	73	32.4
Number of children (n = 227)		
1	126	55.5
2	77	33.9
3	21	9.3
4	2	0.9
5+	1	0.4
Further children planned (n = 223)		
Yes	88	39.5
No	71	31.8
Currently pregnant	13	5.8
Unsure	51	22.9
Marital status (n = 225)		
Single	5	2.2
Partnered	39	17.3
Married	180	80
Divorced	1	0.4
Household income ($AUD) (n = 219)		
Less than $25 000	1	0.5
$25 000–$49 999	5	2.3
$50 000–$69 999	6	2.7
$70 000–$99 999	27	12.3
$100 000–$129 999	46	21
$130 000–$149 999	35	16
More than $150 000	99	45.2
Political affiliation (n = 214)		
Liberal/left	97	45.4
Moderate/centrist	86	40.2
Conservative/right	31	14.5
Has a disability (n = 226)		
Yes	3	1.3
No	223	98.7
Has close family member with a disability (n = 224)		
Yes	54	24.1
No	170	75.9

*The proportion of women who received any high-risk result was 2.2%, comparable to the overall number of high-risk results from the *percept* test (2.1%, internal VCGS data).

†All confirmed with invasive diagnostic testing; all three pregnancies were terminated.

‡False positive.

### Mixed support for conditions/traits identifiable with current testing

**Figure 1:**
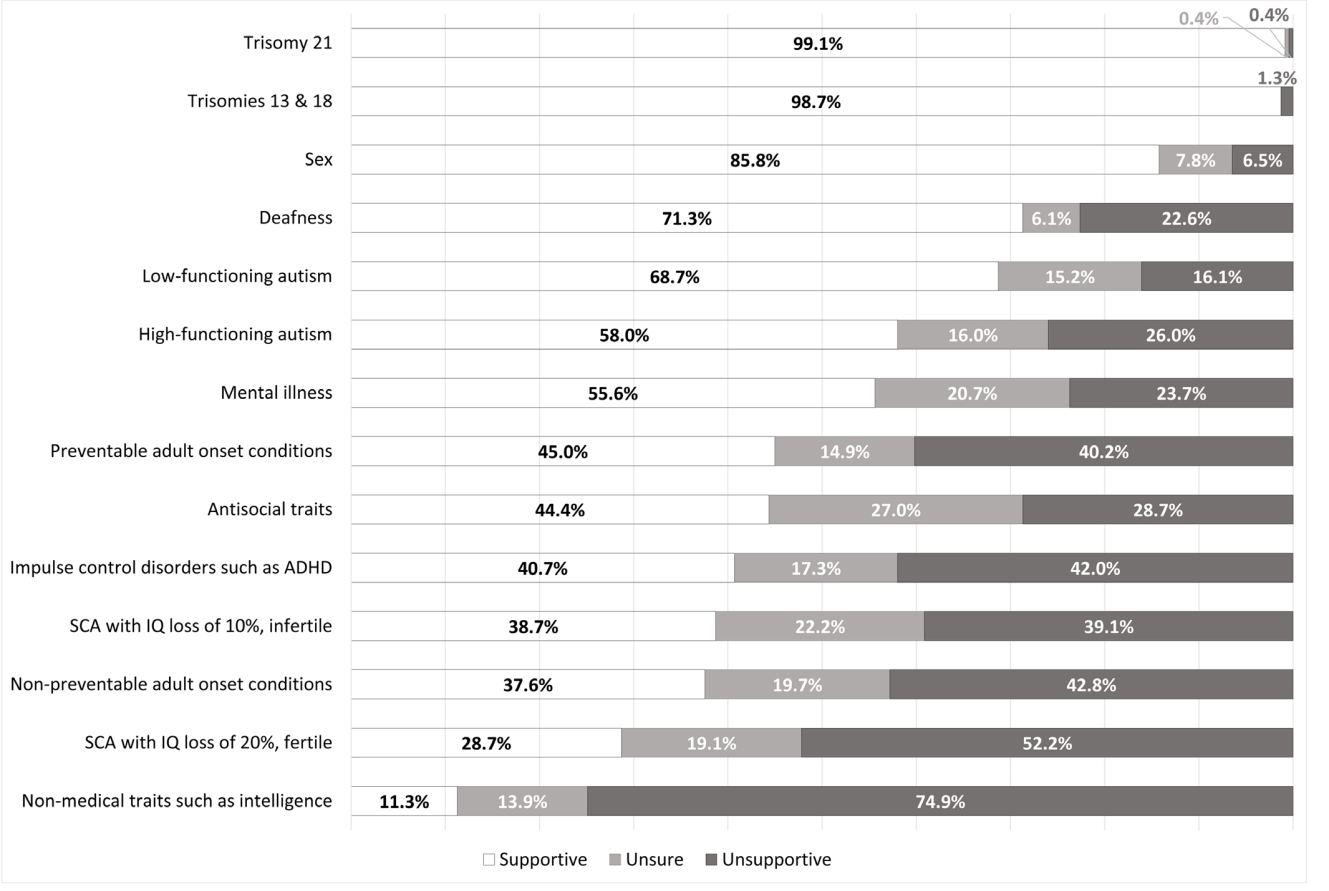
Attitudes towards the availability of NIPT for a particular trait or condition. NIPT, non-invasive prenatal testing; SCA, sex chromosome aneuploidy; ADHD, attention deficit hyperactivity disorder; IQ, intelligence quotient.

**Figure 2:**
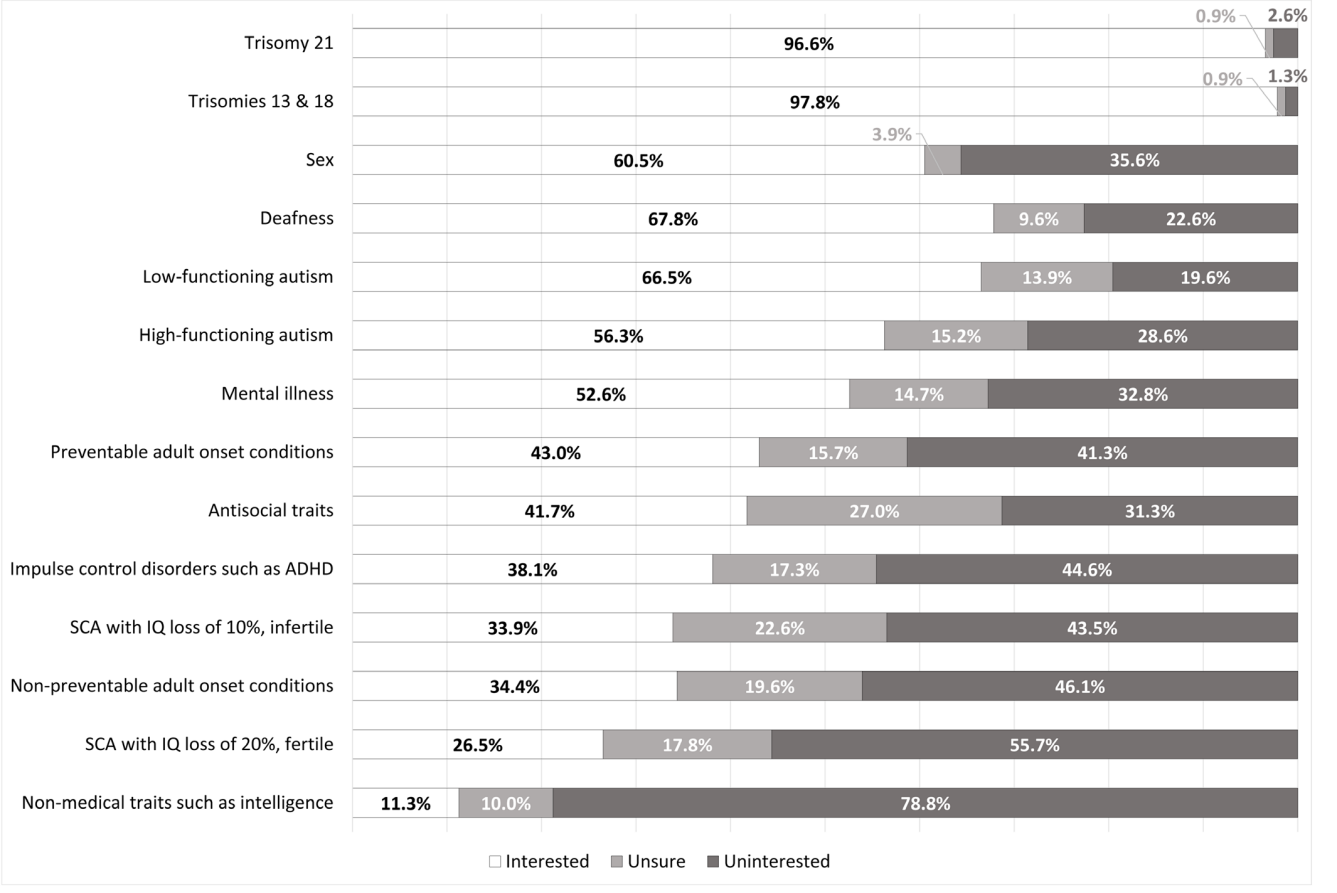
Personal interest in using NIPT for a particular trait or condition. NIPT, non-invasive prenatal testing; SCA, sex chromosome aneuploidy.

General support was found for most conditions/traits identifiable with current NIPT. Most respondents supported availability of NIPT to determine sex (199/232, 85.8%, figure 1), although fewer indicated a personal interest in using NIPT to do so (141/233, 60.5%, figure 2). Termination of pregnancy (TOP) based on sex was viewed negatively, with no respondents indicating that they would consider it and all but two of the respondents (231/233, 99.1%) indicating they would ‘Definitely not’ undergo TOP based on sex. Thirty-one per cent of respondents (73/235) indicated that determination of fetal sex was a reason they underwent NIPT.

**Figure 3:**
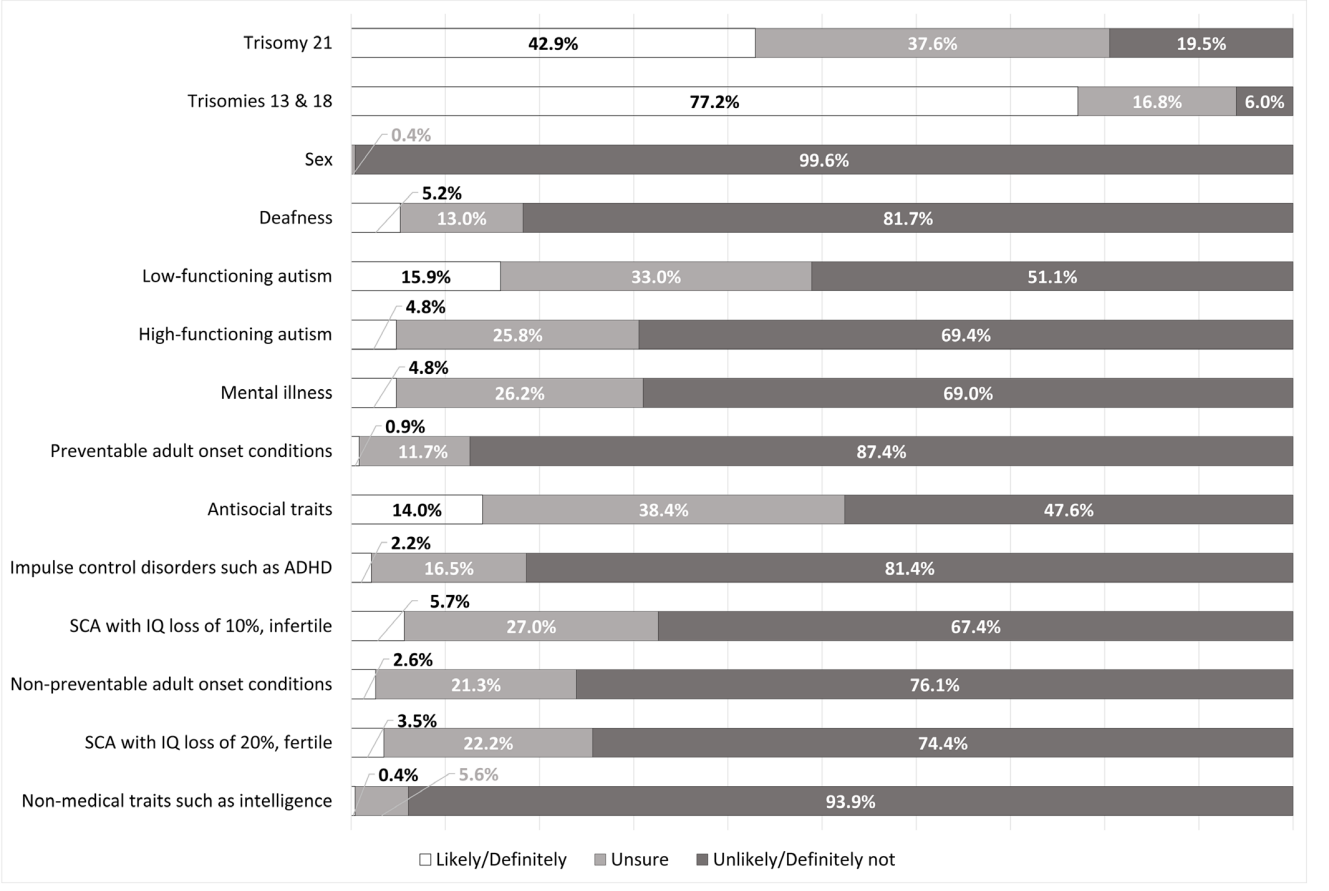
Likelihood of terminating a pregnancy on the basis of a particular trait or condition. SCA,  sex chromosome aneuploidy.

Almost every respondent supported NIPT availability for trisomy 21 screening (231/233, 99.1%, figure 1), and similarly indicated an interest in using it for this purpose (225/233, 96.6%, figure 2). A sizeable minority (97/226, 42.9%, figure 3) indicated they would consider TOP for trisomy 21.

Results for questions regarding trisomies 13 and 18 had similarly high levels of support, with 98.7%% (230/233, figure 1) believing NIPT should be available for trisomies 13 and 18. However, respondents were more interested in using NIPT (226/231, 97.8%, figure 2) and were significantly more likely to have indicate they would consider TOP for these conditions (179/232, 77.2%, p<0.00001, figure 3) than for trisomy 21. Three respondents indicated they had received a high-risk result for trisomy 21, 13 or 18, confirmed by invasive testing. All three underwent TOP.

There was comparatively less interest in screening for the SCAs, which are currently included on the test panel. For both SCA options, responses tended towards less supportive views for all three questions. However, respondents were more likely to believe in availability (p=0.00002), be willing to use NIPT (p=0.002) and indicate a willingness to consider TOP (p=0.04) for an SCA resulting in infertility with a lesser effect on intelligence. One respondent received a high-risk result for an SCA but did not undergo further invasive testing; she indicated it was determined postnatally to be a false positive.

### Responses to scenarios that are not currently tested by NIPT

Respondents had supportive views towards availability of NIPT for deafness (164/230, 71.3%, figure 1) and were interested in testing for it (156/230, 67.8%, figure 2), but there were generally negative views towards TOP for deafness (188/230, 81.7%, figure 3).

There was interest in using NIPT for some psychiatric and neurodevelopmental disorders. Much of the sample supported availability of NIPT to screen for mental illness (129/232, 55.6%, figure 1) and expressed an interest in using NIPT for this purpose (122/232, 52.6%, figure 2). However, a larger proportion (158/229, 69%, figure 3) had negative views towards considering TOP based on mental illness.

The majority believed NIPT should be available (158/230, 68.7%, figure 1) and were interested in using NIPT (153/230, 66.5%, figure 2) for low-functioning autism. A minority of respondents (36/227, 15.9%, figure 3) indicated that they would consider TOP based on low-functioning autism. This is third on the list of conditions where TOP would be considered after the trisomy disorders. As with low-functioning autism, respondents indicated generally supportive views on availability (134/231, 58%, figure 1) and willingness to use NIPT (130/231, 56.3% figure 2) for high-functioning autism, although to a significantly lesser extent compared with low-functioning autism (p<0.0001 for both). There were significantly higher negative views on TOP (159/229, 69.4%, figure 3) for high-functioning autism when compared with low-functioning autism (p<0.0001).

Although there was some interest in testing for adult-onset conditions, this was lower compared with other groups of traits and conditions. There was a higher rate of negative views for non-preventable adult-onset conditions compared with preventable ones. Most respondents indicated that they were unlikely to consider TOP for both groups of conditions, although the rate of negative views was higher in relation to TOP for preventable than non-preventable adult-onset conditions (p=0.00001, figure 3). However, in open-ended responses, multiple respondents (n=12) listed several adult-onset conditions that NIPT should be available to test for. There was a focus on neurodegenerative conditions such as Huntington disease, multiple sclerosis, motor neuron disease and Parkinson disease.

In terms of psychiatric and neurodevelopmental disorders, there was less interest in screening for impulse control disorders such as ADHD (p<0.0001) and antisocial traits (highest p value of 0.017) than either autism or mental illness. Respondents were generally not interested in TOP based on impulse control disorders. They also lacked interest towards TOP based on antisocial traits, but to a significantly lesser extent than for impulse control disorders (p<0.0001), with 14% (32/229, figure 3) responding they would consider TOP based on the presence of antisocial traits. This proportion is fourth overall in levels of interest as a basis for TOP, comparable to low-functioning autism.

There was the least amount of interest in testing for non-medical traits such as intelligence, with an overwhelming negative response to all three questions. Only one respondent indicated they would consider TOP on this basis (1/231, 0.4%, figure 3) and 79.2% of respondents (183/231) specifically indicated that they would ‘definitely not’ do so. In text responses, several respondents (n=7) indicated NIPT should specifically not be available for traits such as height and eye, skin and hair colour.

### Comments on general criteria for testing

#### Accuracy and certainty

Multiple respondents highlighted the uncertainty of NIPT results as an important factor in whether NIPT should be available to test for something, as well as emphasising the importance of a definitive result.

*‘Accuracy of test results is key.’* (#55)

*‘I think it should only be used for conditions that can be identified with some certainty on genetic testing.’* (#21)

#### Impact on quality of life

Several respondents highlighted both clinical severity of the condition and impact on the child’s quality of life as well as the impact it could have on the parents’ lives and their capacity to care for the child.

*
‘I feel this test should only be for severe disabilities, resulting in birth defects, stillborn, extremely poor quality of life for child, and if the birth of the child would result to extreme hardship for the parents.’* (#195)

#### Early intervention

Early diagnosis and opportunities for early intervention were seen as very useful possibilities for NIPT.

*
‘I am more strongly in favour of NIPT due to the value I see in early intervention. While difficult to measure I do believe strongly in plasticity in young children and thus in the ability to encourage certain outcomes if a diagnosis is made early.’* (#53)

#### Issues with testing for adult-onset conditions

Although multiple respondents mentioned neurodegenerative conditions such as Huntington disease as possible targets for screening, several respondents expressed concerns possibly underlying the lower level of interest in testing for adult-onset conditions seen in the quantitative data.

*
‘I don’t think it should be available for conditions with typical onset beyond childhood…Late onset conditions give no consideration of medical advancement between NIPT and actual diagnosis which could be 70+years.’* (#55)

*
‘if we do have…full understanding of every condition that our child may have an increased risk of - would this change the way I parent? And could it become a self fulfilling prophecy? Sometimes, is it better not to know certain things, so your children all grow up being treats* (sic) *equally.’*(#140)

#### Designer babies and ‘too much’ testing

Numerous respondents highlighted anxieties around expanding screening to encompass too many traits and encouraging ‘designer babies’, particularly in the context of non-medical traits.

*
‘NIPT risks entering into the unethical (I think) territory of enabling the selection for designer babies and encouraging terminations of people (babies) who would have otherwise potentially lived fulfilling lives.’* (#78)

*‘Too much testing is dangerous. How much genetic information should an individual be able to have on an unborn child?’* (#200)

#### Use of information provided by testing

Decision-making around TOP was not seen as a natural result of undergoing NIPT. There was higher interest for undergoing testing than undergoing TOP for all traits and conditions. Respondents highlighted the value that information can have before the birth of the child.

*
‘Information enables parents to arm themselves with the latest research and ensure their home is a welcoming one for their baby and any special needs.’* (#122)

### Associations with religious and ethical views

There was a significant negative association between religiosity and favourable views on terminating a pregnancy for both trisomy 21 (OR of 0.52, p<0.0001) and trisomies 13 and 18 (OR of 0.63, p<0.0001). There was no significant association between ethical views as measured by the OUS and attitudes towards NIPT. Demographic variables also exhibited no significant associations (see online supplementary material).

## Discussion

This study explored the attitudes of women who had previously used NIPT regarding current and future uses of the technology. These results suggest that women are more interested in screening for medical conditions with onset earlier in life. Respondents were also very concerned about impact on quality of life and test accuracy.

Respondents’ views were most favourable towards the availability and use of NIPT for trisomies 21, 13 and 18 (figures 1 and 2). Women were also most likely to indicate they would consider TOP for these conditions (figure 3). Higher support for testing and TOP for trisomies 13 and 18 is likely due to their severity and lethality. The negative association between religiosity and favourable views to TOP based on the trisomy disorders that is not seen with the other traits and conditions may be influenced in part by familiarity; there is generally higher reported familiarity with the trisomy disorders rather than, for example, SCAs.^22^ This impact of familiarity on attitudes towards prenatal testing may also have been influenced by the fact that respondents indicating that they either identified as having a disability (1.3%) or had a close family member who has a disability (24.1%) were a minority. It is possible that those who have personal experience with disability as well as NIPT may have different perspectives on this approach to prenatal testing.

The proportion of respondents indicating they would consider TOP for trisomy 21 was 43% (figure 3). This is lower than actual termination rates. In a Western Australian study, the rate of TOP on prenatal diagnosis of trisomy 21 was 93%, and this was stable from 1980 to 2013.^23^ Our sample may have been particularly averse to TOP, although a more likely explanation is that women’s attitudes change after receiving a high-risk result (which applies to few respondents here). A 2012 review found that a willingness to undergo TOP was lowest in prospective parents in the general population (23%–33%), higher in pregnant women at high-risk of trisomy 21 (46–86%) and highest in women who had received a definitive prenatal diagnosis of trisomy 21 (89%–97%).^24^

Most respondents supported availability of NIPT for sex determination, and many indicated they themselves would use it for this purpose. Almost one-third of respondents (31%) had used NIPT for this exact reason. Respondents were near-unanimous against considering sex-selective TOP, suggesting other motivations for sex determination. Objections to the use of NIPT for sex determination relate primarily to concerns that it will be used for sex-selective TOP, which underly the Nuffield Council recommendation against NIPT sex determination.^7^ These results suggest this is not the case in this population.

The results regarding the SCAs, where the differences were explicitly noted in terms of fertility and intelligence, suggest that women may to some degree value fertility over intelligence. This decreased valuation of intelligence is in line with responses to the questions on non-medical traits such as intelligence. Respondents did not have favourable views towards these questions and also highlighted testing for physical traits as a concern, as seen in other studies. A 2014 study on the perspectives of women on broadening the scope of NIPT indicated anxiety in relation to the use of NIPT for physical traits such as a ‘crooked nose’.^18^ A 2015 survey by the same researchers found similar results, although the examples used were hair and eye colour; 87% had a negative view towards the use of NIPT for these traits.^17^ The Nuffield Council argues against availability of testing for non-medical traits.^7^ In highlighting anxieties around testing for non-medical traits, women are more likely to focus instead on conditions that impact quality of life, with a childhood onset (particularly early death).^18 25^ However, it is interesting to note that the questions about an SCA with a 20% drop in IQ are effectively asking the same question as those asking about intelligence within the normal range. The differences in responses were much higher when the decrease in intelligence could be attributed to chromosomal anomaly. This suggests that the framing of a trait as medical or non-medical may impact attitudes towards screening. However, one limitation is that respondents may have had different perspectives on what would constitute a pathological decrease in intelligence (ie, an intellectual disability). Providing a specific number (10% or 20%) rather than the general description of ‘increased or decreased intelligence’ may also have influenced some respondents to view the former as pathological and the latter as a non-pathological variation.

There was high interest in screening for psychiatric and neurodevelopmental disorders, particularly autism and mental illness. Although there is relatively little in the literature, results from this study concord with a 2014 study of parents with a child with autism. That study found that 57% would be willing to undergo prenatal testing for autism, and 14% would opt for TOP in the event of a prenatal diagnosis in a following pregnancy.^26^ Favourable views on TOP here for low-functioning autism were much lower than those for trisomy 21, although both are associated with intellectual disability and autism is associated with more behavioural disturbances.^27^ How autism is represented is likely to have a key impact on attitudes towards prenatal screening with NIPT. If the respondent viewed autism through a medical model, as was generally represented in our study, it is likely that they will be more positive towards prenatal screening than those who view autism through other lenses, such as the model of neurodiversity.^28^ It is also important to note that ‘low-functioning’, ‘severe’, ‘high-functioning’ and ‘mild’ are labels with disputed utility and applicability.^29^ However, they have been used here to promote general lay understanding. This simplification of a complex phenotype poses an important limitation for this survey.

The levels of interest in testing for preventable adult-onset conditions such as cancer sit somewhere between an American 2014 study (50.6% favourable)^16^ and a 2015 Dutch study (29% favourable).^17^ The respondents to our survey were more interested in testing for preventable adult-onset conditions than non-preventable ones. While they were broadly negative towards TOP based on adult-onset conditions generally, they were less negative towards the idea of TOP for non-preventable adult-onset conditions. This suggests that testing for preventable adult-onset conditions is useful for informational purposes but testing for non-preventable adult-onset conditions would be more likely to be with the possibility of TOP in mind. Respondents were more positive to testing for congenital or early-onset conditions, and highlighted concerns around testing for conditions that appear later in life, with respondent #140 wondering about the risk of a ‘self-fulfilling prophecy’.

In terms of impact on quality of life, the question of who prenatal screening and selective TOP benefits is of interest. In our study, respondents emphasised conditions that involve a low quality of life, and in these cases, attitudes may be ostensibly related to benefits conferred on the future child. However, when TOP is an option, it may be more philosophically coherent to conceptualise prenatal screening as being of benefit to existing persons. For example, the comparatively high interest in undergoing TOP based on antisocial traits may be due to concern for the quality of life of the parents and other family members rather than the child. The same may be true of other conditions or traits, although motivations were not specifically addressed in this research. The nature of the benefit is also useful to consider. Respondents did not see undergoing TOP as the natural consequence of a high-risk result for any trait or condition. Testing for informational purposes to prevent, prepare, mitigate or adapt to a condition was highlighted. This may benefit both the parents and the future child.

Limitations of this study relate primarily to the nature of the sample. Respondents were generally highly educated, relatively wealthy, and had progressive political views (see table 2). In Australia, NIPT including the *percept* test are available only on a user-pays basis (at time of writing, costing AUD$449^30^), also indicating a cohort of higher socioeconomic status. The findings may not be generalisable to other groups of women. Although invitations were sent randomly, there was potential for participation bias by self-selection within the invited group. A separate limitation may have arisen from differences in respondents’ awareness and familiarity between conditions/traits which may have influenced attitudes. The nature of the description of the conditions and traits in the survey may also have presented a limitation, as they have been simplified for the purpose of lay understanding (eg, the use of functioning labels for autism); the complex nature of many of the phenotypes means that this does not necessarily produce a completely accurate picture of the conditions and traits and this may affect responses.

The limited qualitative enquiry highlights the opportunity for further qualitative research to explain the findings. This work could also include other populations, including women who have not used NIPT, men, people with disabilities and healthcare workers.

The findings of this study suggest that women support the availability of NIPT for several traits and conditions which may become possible in the future. There was support for a less restrictive approach than current recommendations such as the Nuffield Council report. However, women do remain concerned about ‘too much’ screening. Moving towards resolution of this tension requires further research to dissect the factors contributing to views of women on NIPT.
