# Supplementary material for: ‘Is it better not to know certain things?’: views of women who have undergone non-invasive prenatal testing on its possible future applications
Source: J Med Ethics. 2019 Jan 24;45(4):231–8. doi: 10.1136/medethics-2018-105167 (PMC6582742; doi:10.1136/medethics-2018-105167)
Supplement: Supplementary file 1 [file medethics-2018-105167supp001.pdf]

Non-invasive prenatal testing: future possibilities

What is your current age?

- ○ Under 18 - you are unfortunately not eligible to complete this survey
- ○ 18-25
- ○ 26-30
- ○ 31-35
- ○ 36-40
- ○ 41+

YOUR EXPERIENCE WITH NIPT

What were your reasons for seeking NIPT (the *percept*™ test)? Please tick all that apply.

- ☐ I wanted to know if a chromosomal abnormality is present in my baby
- ☐ I/baby's father/other family member have a genetic or medical condition
- ☐ I wanted reassurance
- ☐ I wanted to know the sex of the baby
- ☐ I have a risk factor for a chromosomal abnormality such as increased age
- ☐ The medical professional managing my pregnancy advised me to have the test
- ☐ I wanted to avoid a CVS or amniocentesis procedure
- ☐ Other ______________________________________

What year(s) did you undergo NIPT?

- ☐ 2013
- ☐ 2014
- ☐ 2015
- ☐ 2016
- ☐ 2017

<u>Before</u> undergoing NIPT, did you or your partner have (please tick any that apply):

- ☐ Genetic screening (e.g. for cystic fibrosis carrier status)
- ☐ Ultrasound
- ☐ Combined first-trimester screening (blood test and ultrasound to estimate the risk of a baby having a chromosomal abnormality)
- ☐ I don't recall

Where did you get information about NIPT? Please tick all that apply.

- ☐ My doctor
- ☐ My midwife
- ☐ Medical websites
- ☐ Social media and forums
- ☐ Family & friends
- ☐ Other ______________________________________

Who were the primary professionals treating you during your pregnancy?

- o GP alone
- o Midwife alone
- o GP shared care with public hospital obstetric unit
- o Public hospital obstetric unit
- o Private obstetrician
- o Other ______________________________________

Do you feel you were provided with adequate information on the nature of the *percept*™ test and possible results?

Definitely not   Mostly not   Neutral   Mostly   Definitely

Do you feel you were sufficiently informed of what the consequences of a positive result (e.g. for trisomy 21 (Down syndrome)) would be?

Definitely   Mostly   Neutral   Mostly not   Definitely not

After undergoing NIPT, did you undergo other forms of prenatal screening or testing? Please tick all that apply.

- ☐ None
- ☐ Ultrasound
- ☐ Combined first-trimester screening
- ☐ Amniocentesis
- ☐ Chorionic villus sampling (CVS)

Do you feel you were provided with adequate information and counselling after you received the results of the *percept*™ test?

Definitely not | Mostly not | Neutral | Mostly | Definitely

What was the result of your test?

- o No increased risk of any chromosomal abnormality (go to page 8)
- o Increased risk of trisomy 21 (Down syndrome), 13 or 18 (go to page 7)
- o Increased risk of too many or too few sex chromosomes (X or Y) (go to page 7)
- o Other (go to page 7)

Did you have any of the following procedures? Please tick any that apply.

- ☐ Chorionic villus sampling (CVS)
- ☐ Amniocentesis
- ☐ Neither (go to page 8)

If either amniocentesis or CVS, what was the result?

- o Typical chromosome result
- o Trisomy 21, 13, or 18
- o Increased (XXX, XXY, XYY) or decreased (X) sex chromosome number
- o Other

If a chromosomal abnormality was identified, did you:

- o Continue the pregnancy
- o Have a pregnancy termination

If you had another pregnancy, how likely would you be to undergo NIPT again?

Definitely not	Unlikely	Unsure	Likely	Definitely

Do you think termination of a pregnancy (abortion) should be legal and accessible?

- o Yes
- o No
- o It depends ___________________________________

Would you personally consider a termination of a pregnancy for any reason?

Definitely not	Probably not	Unsure	Probably	Definitely

CURRENT & FUTURE USES OF NIPT

Many of the following are not possible now but may be in the future. We are interested to know what you think about these uses of NIPT and whether you think these should be offered in the future.

We understand that some of the following questions might be confronting or of a sensitive nature. Once again, all of your answers to this survey are *anonymous*, and we appreciate your responses greatly.

Non-invasive prenatal tests such as *percept™* already test for the sex of the baby. You can choose whether or not you would like to know this.

Do you think NIPT should be available to find out the *sex* in a pregnancy?

Definitely	Probably	Unsure	Probably not	Definitely not

Would you want to use NIPT to find out the *sex* in your pregnancy?

Definitely not	Probably not	Unsure	Probably	Definitely

How likely would you be to terminate based on the *sex* of your baby?

Definitely not	Unlikely	Unsure	Likely	Definitely

Trisomy 21, or Down syndrome, is a non-lethal genetic condition caused by an extra copy of chromosome 21. People with Down syndrome have physical growth delays, intellectual disability, and are more prone to heart defects. With the right support, many people with Down syndrome live fulfilling lives.

Do you think NIPT should be available to find out if a baby had a *non-lethal genetic condition*, such as trisomy 21 (Down Syndrome)?

Definitely not Probably not Unsure Probably Definitely

Would you want to use NIPT to find out if your baby had a *non-lethal genetic condition*, such as trisomy 21 (Down Syndrome)?

Definitely Probably Unsure Probably not Definitely not

How likely would you be to terminate based on a *non-lethal genetic condition*, such as trisomy 21 (Down Syndrome)?

Definitely not Unlikely Unsure Likely Definitely

Trisomy 13 (Patau syndrome) and trisomy 18 (Edwards syndrome) are genetic conditions caused by extra copies of chromosomes 13 and 18, respectively. They are associated with severe physical and intellectual disabilities. Most babies with these conditions are either stillborn or die soon after birth.

Do you think NIPT should be available to find out if a baby had a condition that was *generally fatal in the early weeks of life*, such as trisomy 13 or 18?

Definitely Probably Unsure Probably not Definitely not

Would you want to use NIPT to find out if your baby had a condition that was *generally fatal in the early weeks of life*, such as trisomy 13 or 18?

Definitely not Probably not Unsure Probably Definitely

How likely would you be to terminate based on a condition that was generally *fatal in the early weeks of your baby's life*, such as trisomy 13 or 18?

Definitely Likely Unsure Unlikely Definitely not

Mental illness can sometimes run in families. In these cases, there can be strong genetic risk factors for certain mental illnesses. These can include conditions such as depression, bipolar disorder, and schizophrenia.

Do you think NIPT should be available to find out if a baby had an increased risk of *mental illness*?

Definitely not   Probably not   Unsure   Probably   Definitely

Would you want to use NIPT to find out if your baby had an increased risk of *mental illness*?

Definitely   Probably   Unsure   Probably not   Definitely not

How likely would you be to terminate based on an increased risk of *mental illness*?

Definitely not   Unlikely   Unsure   Likely   Definitely

There is not a specific gene for non-medical traits such as increased intelligence, but rather many genes contribute to these traits. Studies may be able to identify genetic patterns *associated* with these traits in the future.

Do you think NIPT should be available to find out about *non-medical traits*, such as increased or decreased intelligence?

Definitely not   Probably not   Unsure   Probably   Definitely

Would you want to use NIPT to find out about *non-medical traits*, such as increased or decreased intelligence?

Definitely   Probably   Unsure   Probably not   Definitely not

How likely would you be to terminate based on *non-medical traits*, such as increased or decreased intelligence?

Definitely   Likely   Unsure   Unlikely   Definitely not

There is no single cause of autism spectrum disorders, but there is a strong genetic basis for them. Autism spectrum disorders are characterised by difficulties with social interaction, difficulties with communication, and repetitive behaviours.

Autism spectrum disorders are classified from mild impact (sometimes called Asperger syndrome) through to severe autism, which is associated with a severe impact on intellect. There are no genetic tests that can currently diagnose most autism spectrum disorders, but this may change in the future.

Do you think NIPT should be available to find out about conditions such as *low-functioning/severe autism*?

Definitely   Probably   Unsure   Probably not   Definitely not

Would you want to use NIPT to find out about conditions such as *low-functioning/severe autism*?

Definitely   Probably   Unsure   Probably not   Definitely not

How likely would you be to terminate based on conditions such as *low-functioning/severe autism*?

Definitely   Likely   Unsure   Unlikely   Definitely not

Do you think NIPT should be available to find out about conditions such as ***high**-functioning/mild autism* (Asperger Syndrome)?

Definitely not | Probably not | Unsure | Probably | Definitely

Would you want to use NIPT to find out about conditions such as ***high**-functioning/mild autism* (Asperger Syndrome)?

Definitely not | Probably not | Unsure | Probably | Definitely

How likely would you be to terminate based on conditions such as ***high**-functioning/mild autism* (Asperger Syndrome)?

Definitely not | Unlikely | Unsure | Likely | Definitely

Impulse control disorders are characterised by difficulty in controlling behaviour or resisting an urge. Attention Deficit Hyperactivity Disorder (ADHD) is one such condition. People with ADHD may also have problems paying attention to tasks and this affects their education and employment prospects if left untreated.

Do you think NIPT should be available to find out about *impulse control disorders*, such as ADHD?

Definitely not   Probably not   Unsure   Probably   Definitely

Would you want to use NIPT to find out about *impulse control disorders*, such as ADHD?

Definitely   Probably   Unsure   Probably not   Definitely not

How likely would you be to terminate based on *impulse control disorders*, such as ADHD?

Definitely not   Unlikely   Unsure   Likely   Definitely

Sociopathy is an informal term for Antisocial Personality Disorder (ASPD). People with this disorder generally show a disregard for others, lack of conscience and remorse, and impulsive and aggressive behaviour. This disorder is very difficult to treat.

Do you think NIPT should be available to find out about *antisocial traits* and disorders such as sociopathy?

Definitely Probably Unsure Probably not Definitely not

Would you want to use NIPT to find out about *antisocial traits* and disorders such as sociopathy?

Definitely not Probably not Unsure Probably Definitely

How likely would you be to terminate based on *antisocial traits* and disorders such as sociopathy?

Definitely not Unlikely Unsure Likely Definitely

Some sex chromosome disorders result in decreased general intelligence (as commonly measured by IQ) but they have no effect on fertility. That is, someone with such a disorder can still have their own children.

Do you think NIPT should be available to find out about sex chromosome disorders which *reduce IQ by 20%* but have *no effect on fertility*?

Definitely not   Probably not   Unsure   Probably   Definitely

Would you want to use NIPT to find out about sex chromosome disorders which *reduce IQ by 20%* but have *no effect on fertility*?

Definitely   Probably   Unsure   Probably not   Definitely not

How likely would you be to terminate based on sex chromosome disorders which *reduce IQ by 20%* but have *no effect on fertility*?

Definitely   Likely   Unsure   Unlikely   Definitely not

Some sex chromosome disorders generally result in infertility - people with these conditions will be unable to have children. They may also result in slightly decreased general intelligence (as commonly measured by IQ).

Do you think NIPT should be available to find out about sex chromosome disorders which *reduce IQ by 10%* but *cause infertility*?

Definitely not Probably not Unsure Probably Definitely

Would you want to use NIPT to find out about sex chromosome disorders which *reduce IQ by 10%* but *cause infertility*?

Definitely Probably Unsure Probably not Definitely not

How likely would you be to terminate based on sex chromosome disorders which *reduce IQ by 10%* but *cause infertility*?

Definitely not Unlikely Unsure Likely Definitely

Deafness is sometimes genetic.

Do you think NIPT should be available to find out about traits such as *deafness?*

Definitely Probably Unsure Probably not Definitely not

Would you want to use NIPT to find out about traits such as *deafness?*

Definitely not Probably not Unsure Probably Definitely

How likely would you be to terminate based on traits such as *deafness?*

Definitely Likely Unsure Unlikely Definitely not

Many diseases that appear later in life have a genetic basis. These include heart disease and cancer. However, preventive and screening measures can help avert or reduce these diseases (for example, a healthy diet, or a regular colonoscopy to check for signs of bowel cancer in a person with an increased risk of this condition).

Do you think NIPT should be available to identify a baby's risk for *adult onset diseases for which preventive and/or screening measures exist*, such as heart disease or cancer?

Definitely not   Probably not   Unsure   Probably   Definitely

Would you want to use NIPT to identify your baby's risk for *adult onset diseases for which preventive and/or screening measures exist*, such as heart disease or cancer?

Definitely not   Probably not   Unsure   Probably   Definitely

How likely would you be to terminate based on your baby's risk for *adult onset diseases for which preventive and/or screening measures exist*, such as heart disease or cancer?

Definitely not   Unlikely   Unsure   Likely   Definitely

Alzheimer disease can sometimes be due to an identifiable gene fault and this often manifests earlier in life than typical Alzheimer disease, often before the age of 65. People with Alzheimer disease experience dementia and generally die three to ten years from onset. No definitive preventive measures currently exist.

Do you think NIPT should be available to identify a baby's risk for *adult onset diseases for which **no** preventive measures currently exist*, such as early-onset Alzheimer disease?

Definitely Probably Unsure Probably not Definitely not

Would you want to use NIPT to identify your baby's risk for *adult onset diseases for which **no** preventive measures currently exist*, such as early-onset Alzheimer disease?

Definitely Probably Unsure Probably not Definitely not

How likely would you be to terminate based on your baby's risk for *adult onset diseases for which **no** preventive measures currently exist*, such as early-onset Alzheimer disease?

Definitely Likely Unsure Unlikely Definitely not

Are there any other conditions you think NIPT *should* be available to test for?

Are there any other conditions you think NIPT *should not* be available to test for?

Do you think NIPT should be (please select one):

- Only available on a user pays basis, as is currently the case
- Government funded (e.g. by Medicare)
- Government funded for significant medical conditions, but user pays for non-medical traits such as intelligence within the standard range

Do you think it is fair that women/couples currently must pay to access NIPT?

Definitely not Probably not Unsure Probably Definitely

PERSONAL BELIEFS

These questions are unrelated to your experience of NIPT but may help us understand how your answers to questions relate to your beliefs.

Please select the most applicable to you:

How often do you think about religious issues?

Never Rarely Occasionally Often Very often

To what extent do you believe that God or something divine exists?

Not at all Not very much Moderately Quite a bit Very much so

How often do you take part in religious services?

Never Rarely Occasionally Often Very often

How often do you pray?

Never Rarely Occasionally Often Very often

How often do you experience situations in which you have the feeling that God or something divine intervenes in your life?

Never Rarely Occasionally Often Very often

People often think about issues such as the use of NIPT in different ways. We would like to look at associations between people's responses to this survey and their general beliefs. This is a new scale that has been developed to ascertain people's approach to these sorts of issues. There are no right or wrong answers!

Please note these are purely imaginary and should reflect what you believe is right, not what is legal or illegal.

Please indicate how much you agree or disagree with each of the following statements

(1 = strongly disagree, 4 = neither agree nor disagree, 7 = strongly agree)

If the only way to save another person's life during an emergency is to sacrifice one's own leg, then one is morally required to make this sacrifice.

1 2 3 4 5 6 7

From a moral point of view, we should feel obliged to give one of our kidneys to a person with kidney failure since we don't need two kidneys to survive, but really only one to be healthy.

1 2 3 4 5 6 7

From a moral perspective, people should care about the well-being of all human beings on the planet equally; they should not favor the well-being of people who are especially close to them either physically or emotionally.

1 2 3 4 5 6 7

It is just as wrong to fail to help someone as it is to actively harm them yourself.

1 2 3 4 5 6 7

It is morally wrong to keep money that one doesn't really need if one can donate it to causes that provide effective help to those who will benefit a great deal.

1 2 3 4 5 6 7

It is morally right to harm an innocent person if harming them is a necessary means to helping several other innocent people.

1 2 3 4 5 6 7

If the only way to ensure the overall well-being and happiness of the people is through the use of political oppression for a short, limited period, then political oppression should be used.

1 2 3 4 5 6 7

It is permissible to torture an innocent person if this would be necessary to provide information to prevent a bomb going off that would kill hundreds of people.

1 2 3 4 5 6 7

Sometimes it is morally necessary for innocent people to die as collateral damage if more people are saved overall.

1 2 3 4 5 6 7

YOUR INFORMATION

What is your highest completed level of education?
- o Primary school
- o Secondary school (VCE or equivalent)
- o Technical or trade certificate
- o Bachelor’s degree
- o Postgraduate qualification (e.g. Masters, PhD)

Do you live in Victoria?
- o Yes
- o No

If yes, what is your postcode? _______________

How many children do you currently have?
- o 0
- o 1
- o 2
- o 3
- o 4
- o 5+

Do you plan to have more children?
- o Yes
- o No
- o Currently pregnant
- o Unsure

What is your marital status?

- ○ Single
- ○ Partnered
- ○ Married
- ○ Divorced
- ○ Other ____

What is your combined household income?

- ○ Less than $25,000
- ○ $25,000 – $49,999
- ○ $50,000 - $69,999
- ○ $70,000 - $99,999
- ○ $100,000 - $129,999
- ○ $130,000 - $149,999
- ○ More than $150,000

In general, how liberal/left-wing or conservative/right-wing do you consider yourself to be?

(1 = Very liberal/left; 4 = Moderate/Centrist; 7 = Very Conservative/right)

1 2 3 4 5 6 7

Do you identify as having a disability?

- ○ Yes
- ○ No

Do any of your close family members (e.g. sibling, child) identify as having a condition or trait mentioned in this survey?

- o Yes
- o No

If yes, which one(s)?

Do any of your close family members (e.g. sibling, child) identify as having any other disability?

- o Yes
- o No

Are there any other points you would like to make in relation to this survey?

Thank you for completing this survey!

Your participation is greatly valued and we appreciate you taking the time to respond.
